# Supplementary material for: Analysis of 4-Hydroxyphenyllactic Acid and Other Diagnostically Important Metabolites of α-Amino Acids in Human Blood Serum Using a Validated and Sensitive Ultra-High-Pressure Liquid Chromatography-Tandem Mass Spectrometry Method
Source: Metabolites. 2023 Nov 3;13(11):1128. doi: 10.3390/metabo13111128 (PMC10673366; doi:10.3390/metabo13111128)
Supplement: Supplementary file 1 [file metabolites-13-01128-s001.zip › Fig. S1-S6_16.10.2023.pdf]

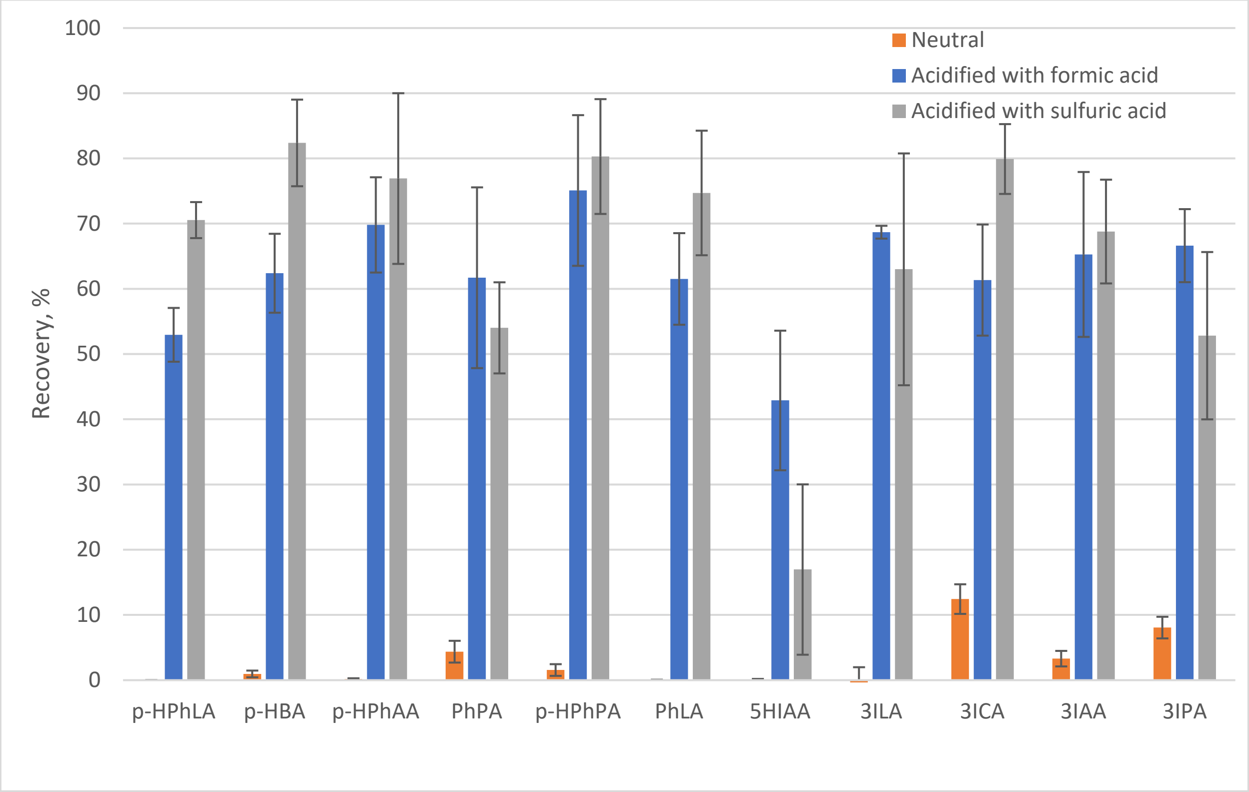

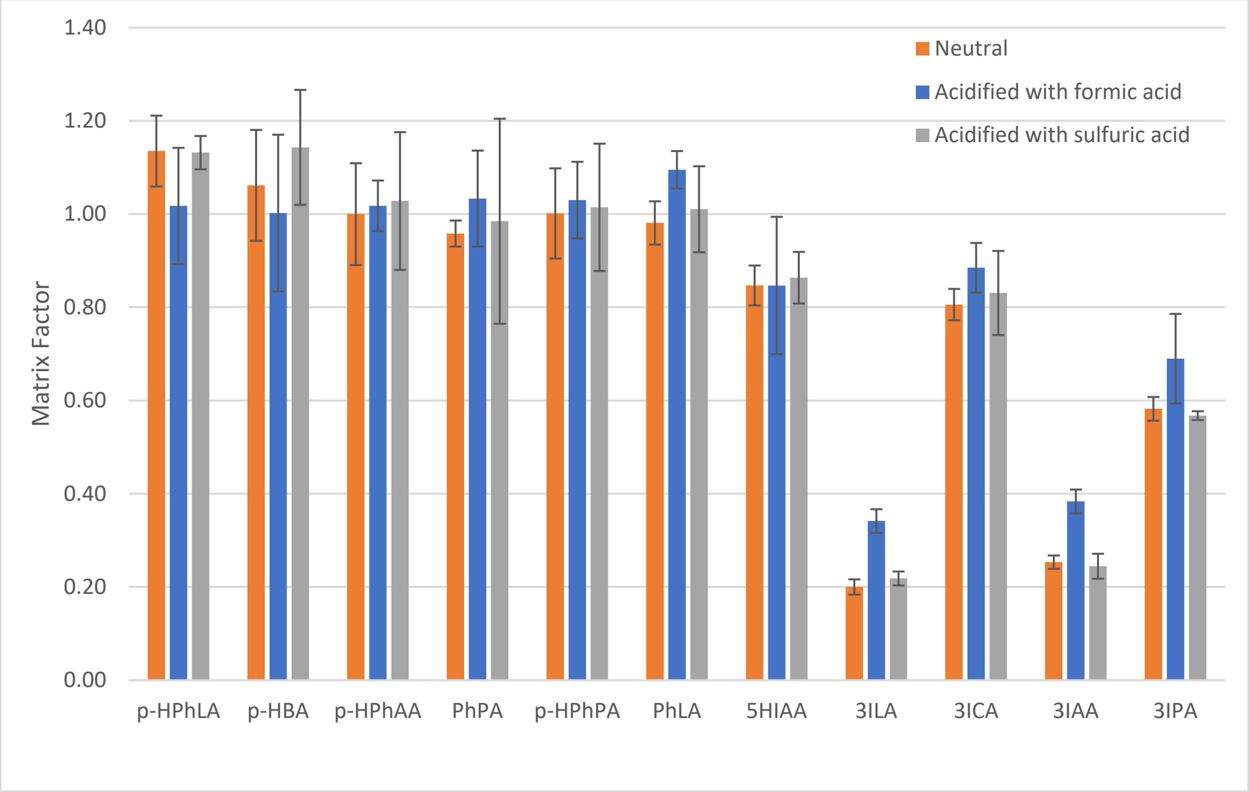

Fig. S1. The influence of pH and type (neutral and acidic pH with formic or sulfuric acids) of acid on the recovery and matrix factor of the analytes using liquid-liquid extraction (n = 3, P = 0.95)

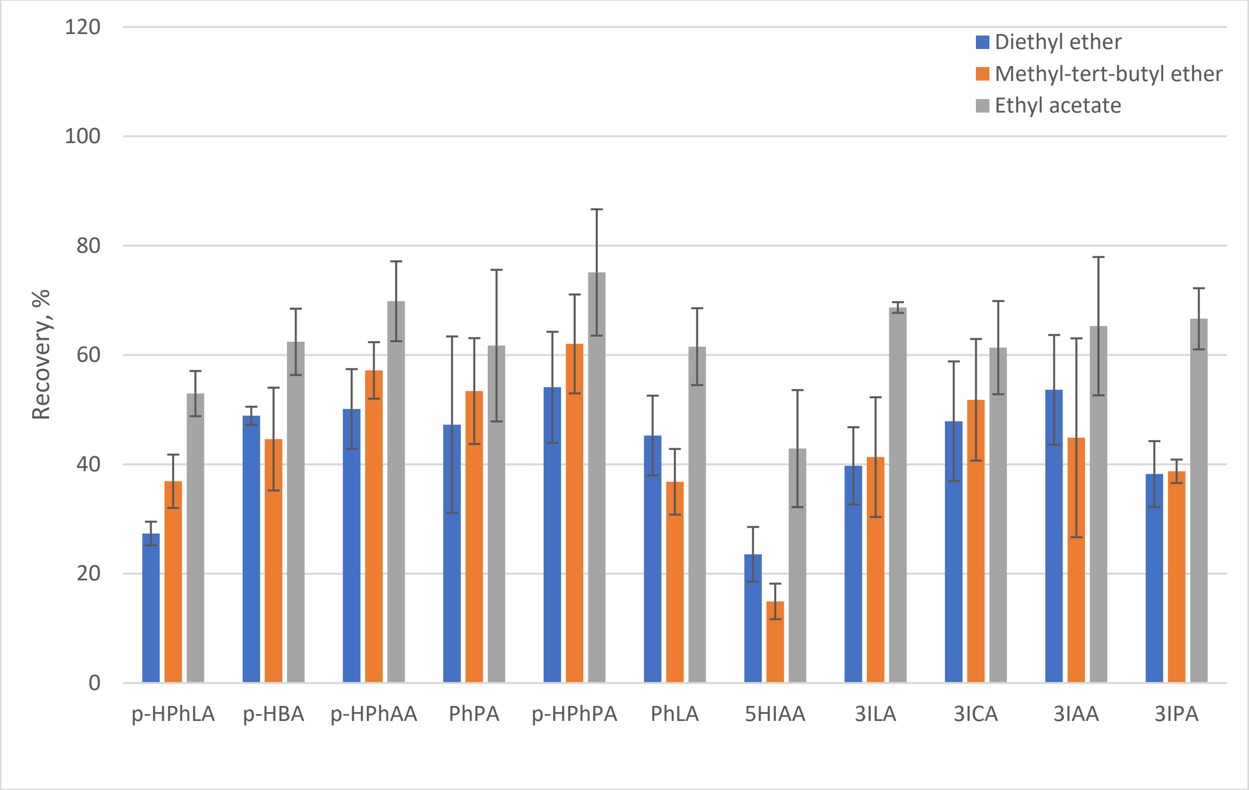

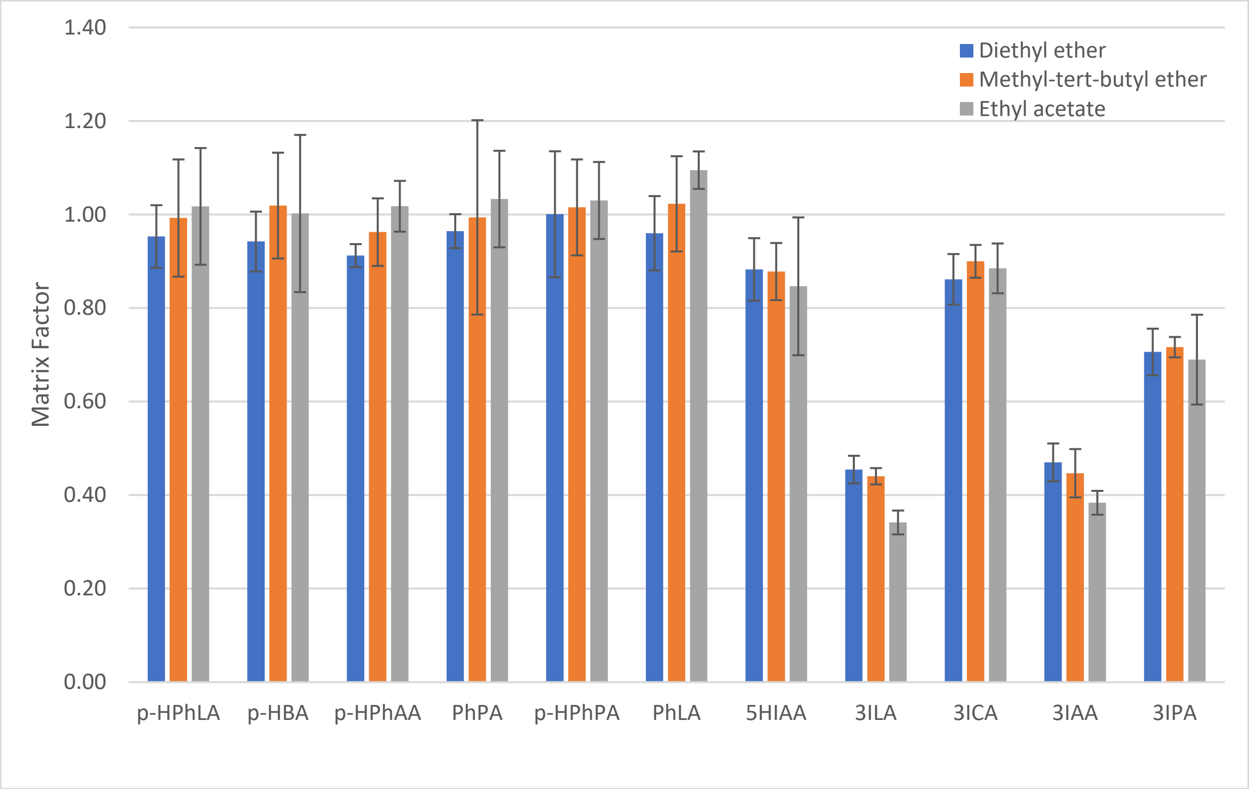

Fig. S2. The influence of the type of the organic extractant (diethyl ether, methyl-tert-butyl ether, and ethyl acetate) on the recovery and matrix factor of the analytes using liquid-liquid extraction (n = 3, P = 0.95)

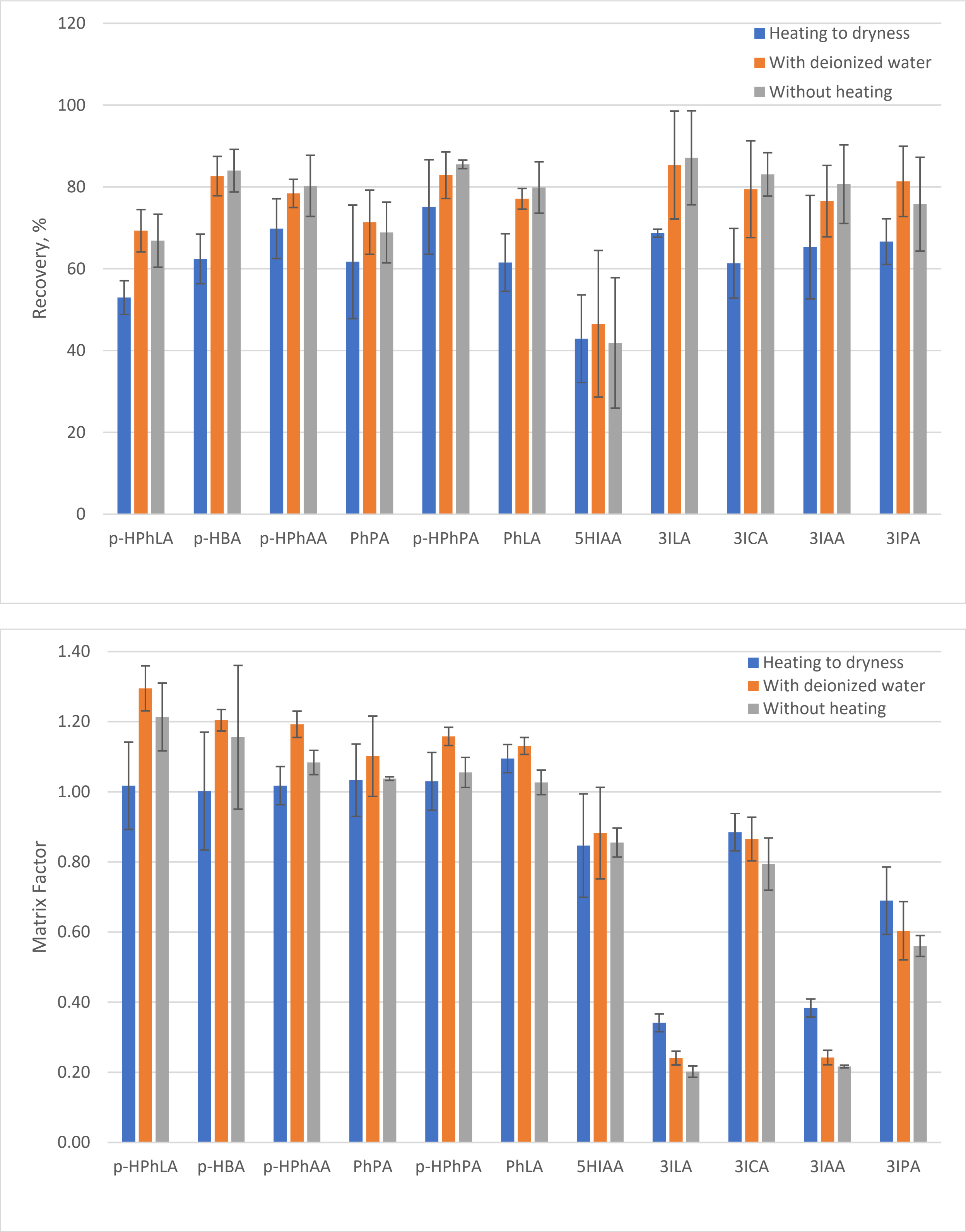

Fig. S3. The influence of the evaporation conditions (heating to dryness, with deionized water, without heating) on the recovery and matrix factor of the analytes using liquid-liquid extraction (n = 3, P = 0.95)

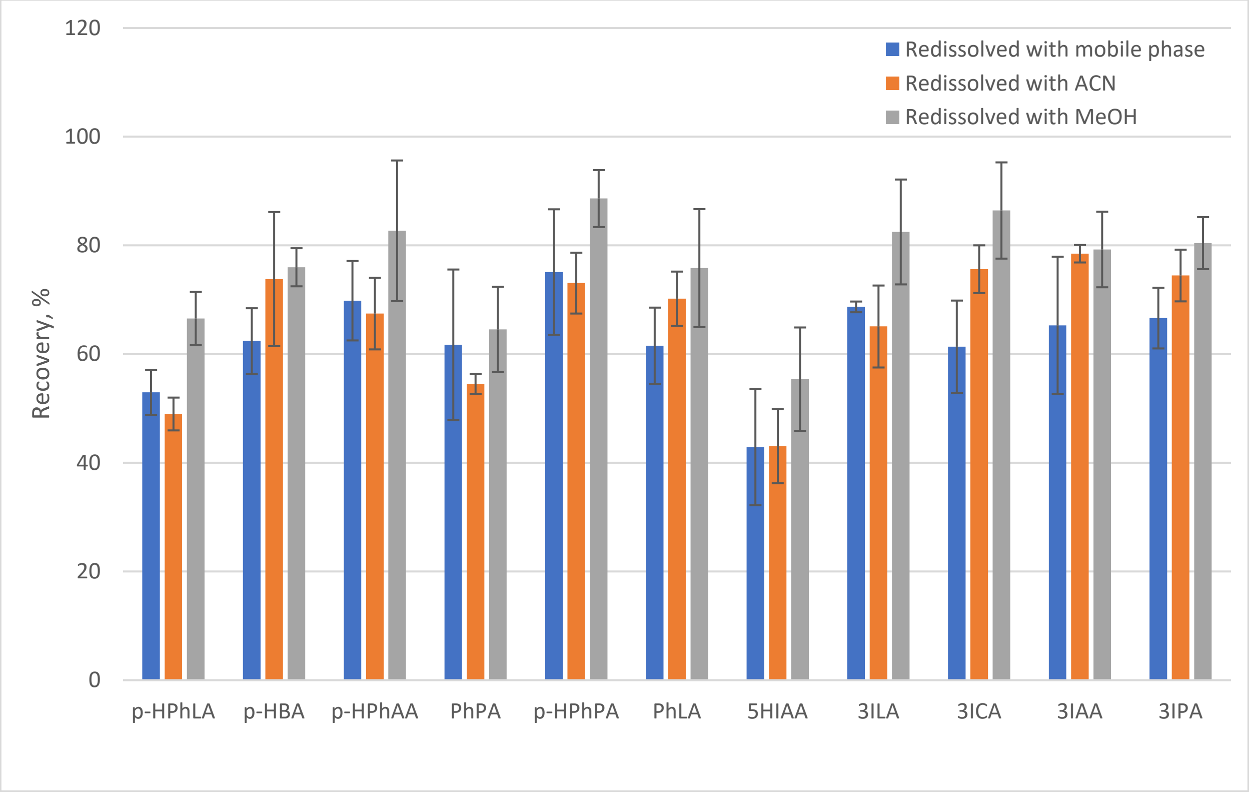

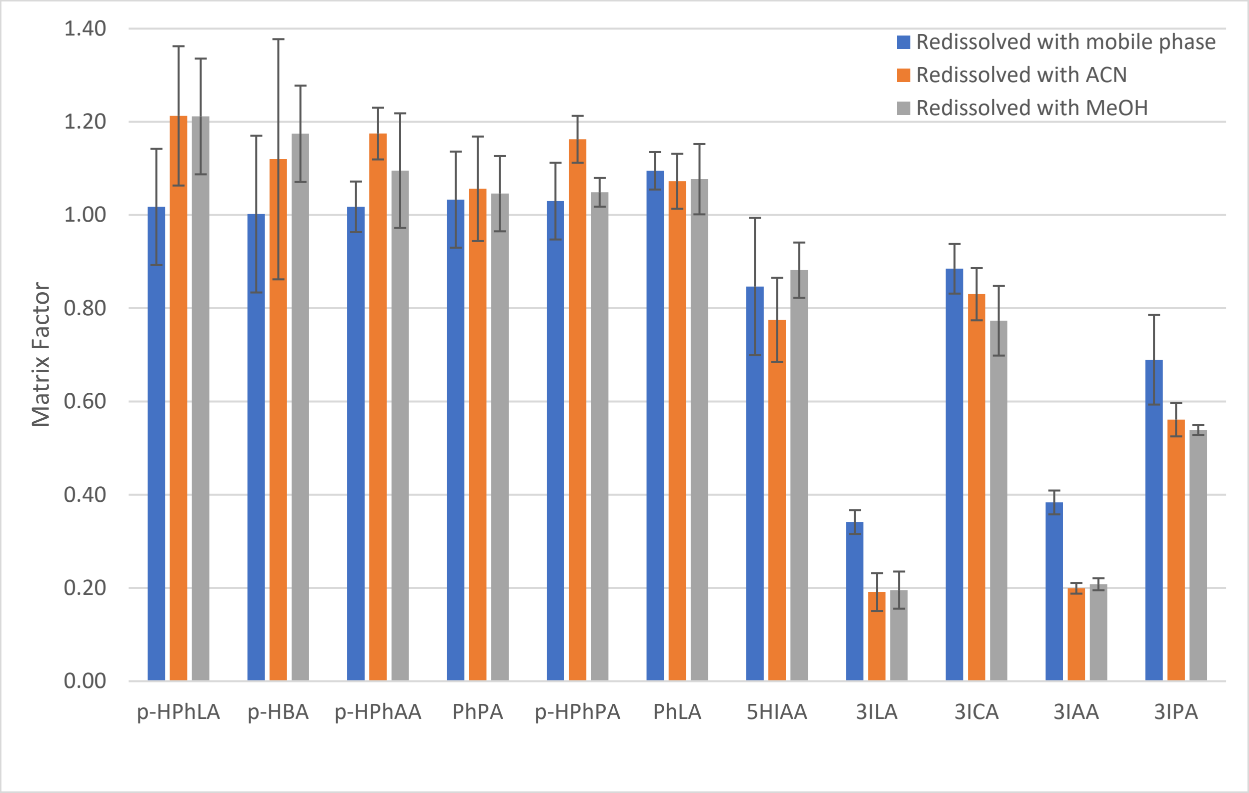

Fig. S4. The influence of the type of the solvent, which was used to resolve the dry residue (the mobile phase, acetonitrile or methanol) on the recovery and matrix factor of the analytes using liquid-liquid extraction (n = 3, P = 0.95)

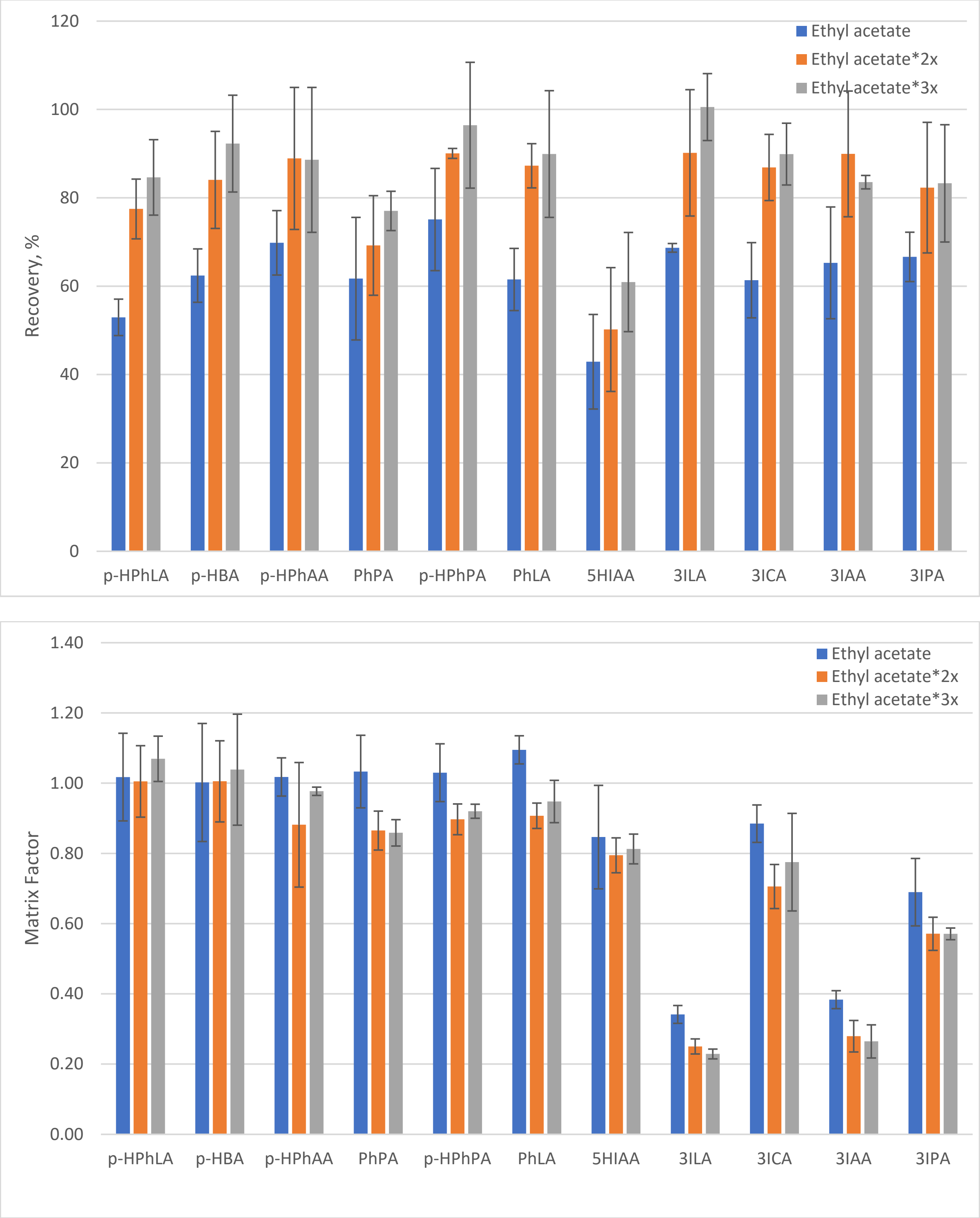

Fig. S5. The influence of the single, double and triple extractions on the recovery and matrix factor of the analytes using liquid-liquid extraction (n = 3, P = 0.95)

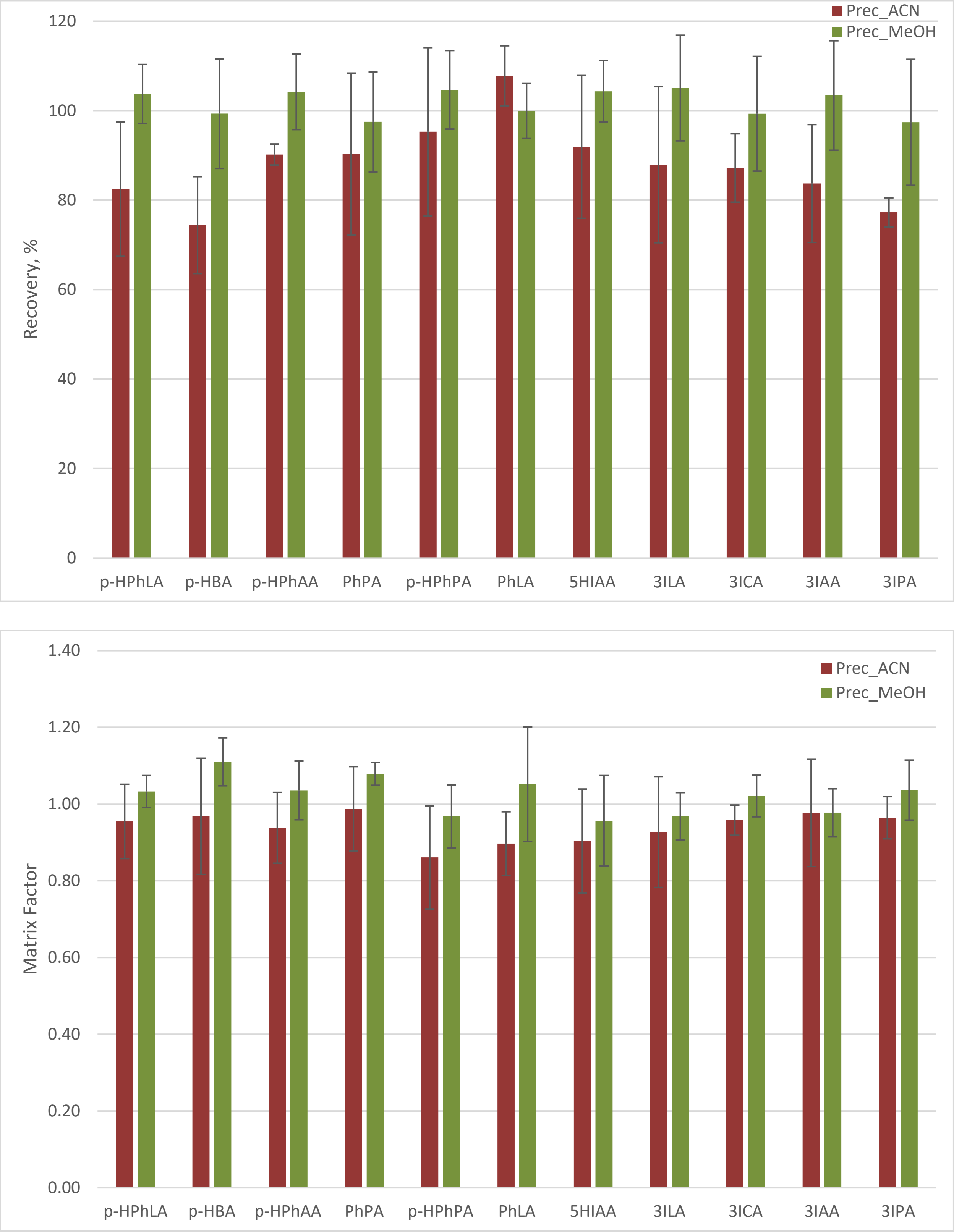

Fig. S6. The influence of the type of organic precipitators (acetonitrile or methanol) on the recovery and matrix factor of the analytes using protein precipitation (n = 3, P = 0.95)
